# Evidence and clinical relevance of maternal-fetal cardiac coupling: A scoping review

**DOI:** 10.1371/journal.pone.0287245

**Published:** 2023-07-12

**Authors:** Thomas J. Nichting, Maretha Bester, Rohan Joshi, Massimo Mischi, Myrthe van der Ven, Daisy A. A. van der Woude, S. Guid Oei, Judith O. E. H. van Laar, Rik Vullings

**Affiliations:** 1 Department of Gynaecology and Obstetrics, Veldhoven, The Netherlands; 2 Department of Electrical Engineering, Eindhoven University of Technology, Eindhoven, The Netherlands; 3 Eindhoven MedTech Innovation Centre, Eindhoven, The Netherlands; 4 Department of Patient Care and Monitoring, Philips Research, Eindhoven, The Netherlands; 5 Department of Biomedical Engineering, Eindhoven University of Technology, Eindhoven, The Netherlands; 6 Nemo Healthcare, Veldhoven, The Netherlands; Kasr Alainy Medical School, Cairo University, EGYPT

## Abstract

**Background:**

Researchers have long suspected a mutual interaction between maternal and fetal heart rhythms, referred to as maternal-fetal cardiac coupling (MFCC). While several studies have been published on this phenomenon, they vary in terms of methodologies, populations assessed, and definitions of coupling. Moreover, a clear discussion of the potential clinical implications is often lacking. Subsequently, we perform a scoping review to map the current state of the research in this field and, by doing so, form a foundation for future clinically oriented research on this topic.

**Methods:**

A literature search was performed in PubMed, Embase, and Cochrane. Filters were only set for language (English, Dutch, and German literature were included) and not for year of publication. After screening for the title and the abstract, a full-text evaluation of eligibility followed. All studies on MFCC were included which described coupling between heart rate measurements in both the mother and fetus, regardless of the coupling method used, gestational age, or the maternal or fetal health condition.

**Results:**

23 studies remained after a systematic evaluation of 6,672 studies. Of these, 21 studies found at least occasional instances of MFCC. Methods used to capture MFCC are synchrograms and corresponding phase coherence indices, cross-correlation, joint symbolic dynamics, transfer entropy, bivariate phase rectified signal averaging, and deep coherence. Physiological pathways regulating MFCC are suggested to exist either via the autonomic nervous system or due to the vibroacoustic effect, though neither of these suggested pathways has been verified. The strength and direction of MFCC are found to change with gestational age and with the rate of maternal breathing, while also being further altered in fetuses with cardiac abnormalities and during labor.

**Conclusion:**

From the synthesis of the available literature on MFCC presented in this scoping review, it seems evident that MFCC does indeed exist and may have clinical relevance in tracking fetal well-being and development during pregnancy.

## 1. Introduction

Although the mother and fetus are physically distinct from each other, their cardiac systems are connected via the placenta to facilitate gas and nutrient exchange for the fetus [1]. Both cardiac systems are constantly adapting in response to external as well as internal stimuli [2].

For example, the mother’s heart rate (HR) is influenced by the environmental temperature and the time of day but also changes in response to her stress levels [2, 3]. Similarly, the fetal HR will be regulated in response to internal triggers, for example, fetal blood oxygen levels [4], as well as external triggers such as lights and sounds sensed through the maternal abdomen [5]. However, since the external environment of the fetus is that of the maternal womb, the fetus also responds to changes in maternal physiology, for example changing maternal stress levels [6]. Moreover, the fetus forms part of the internal environment of the mother, and maternal HR has also been observed to change in response to fetal movement [7]. Researchers have suggested that maternal HR may respond to changes in fetal HR and vice versa–this mutual interaction is referred to as maternal-fetal cardiac coupling (MFCC) [8].

Since Hildebrandt et al. in 1979 first suggested that there may be an interaction between maternal and fetal heartbeats [9], researchers have investigated the potential existence and applications of MFCC [10–12]. Quantifying and understanding the presence, strength, and direction of MFCC is valuable. Not only could assessments of MFCC elucidate gestational cardiac physiology, but such assessments may also offer tools to track fetal development and screen for maternal and fetal complications [13–15].

The potential interaction between maternal and fetal heart rhythms is a complex and not yet clearly defined research field [8, 16]. Although more than 20 research studies have been published on the topic of MFCC, these studies not only employ different methods and study different populations, but also define MFCC differently. Consequently, how to quantify and interpret MFCC remains unclear. Moreover, while clinical relevance is a common aim of research on physiological coupling, results of MFCC analyses are reported without a clear discussion of the potential clinical implications.

Therefore, an exploratory mapping of existing literature–presented in a clinically accessible manner–is a necessary foundation for future clinically motivated research in this field. As MFCC is an area of emerging research, this topic lends itself to a scoping review. A scoping review provides a detailed overview of all research in the field and goes beyond answering a specific question, as is typically the motivation for a systematic review. In this manner, scoping reviews generate findings that help refine research priorities and inform future primary research [17, 18].

With this scoping review, we aim to ascertain the current state of research on MFCC and, in doing so, form a foundation for future clinically oriented research on this topic. To this end, we perform a search of all available research in this field. Thereafter, we synthesize the evolution of the methodologies employed to capture MFCC. Next, we summarize the results to determine whether MFCC exists and, if so, which physiological pathways may regulate MFCC. Finally, we discuss the potential clinical implications of MFCC.

## 2. Methodology

The methodology for this scoping review followed the framework first suggested by Arksey and O’Malley [17] while incorporating further suggestions and insights from Levac et al. [19], Daudt et al. [20], Munn et al. [18], and Peters et al. [21]. The review was reported per the PRISMA guidelines extension for scoping reviews (PRISMA-ScR) [22]. The protocol for this review was preregistered before the literature search and data extraction on Open Science Framework [23].

### 2.1 Search strategies and study selection

The search strategy was developed in consultation with a clinical librarian and can be found in S1 File. Searches were carried out on 27 October 2022 in PubMed, Embase, and Cochrane. No date limits or other filters were applied, but the language was limited to English, Dutch, and German, owing to the language proficiency of the primary authors. Search results were downloaded and systematically sorted using Rayyan QCRI, a platform specifically designed to manage the review process (https://rayyan.qcri.org/welcome). This platform was also utilized to automatically identify and eliminate duplicate studies identified across multiple databases. Citations and references of the included studies were further searched to identify more potential studies. Additionally, a search was performed of all the works published by the researchers of the included studies to identify any further work concerning MFCC.

Studies had to meet certain criteria to be eligible for the review. All studies assessing MFCC–regardless of the coupling method used, gestational age (GA), or the maternal-fetal health condition–which incorporated HR measurements from both the mother and fetus, were allowed for this scoping review. Studies measuring *only* other types of coupling, e.g. coupling between maternal HR and fetal movement, were excluded.

The review process comprised two levels of screening. First, the title and abstract of all the identified literature were screened. Thereafter, a full-text review of studies identified in the first level was carried out to assess eligibility. The review process was carried out independently by two researchers (MB, TN), blinded to each other’s results [19]. After each level of screening, the identified studies were discussed. Disagreement was resolved by discussion. If necessary, an independent researcher was consulted to decide whether an article should be included.

In some cases, research was disseminated first as a conference paper and thereafter as a journal article. In these cases, when everything reported in the conference paper was encompassed in the journal article, the conference paper was excluded. Furthermore, if only a conference abstract was available for a study without an accompanying paper, the abstract was excluded.

## 3. Results

**Fig 1:**
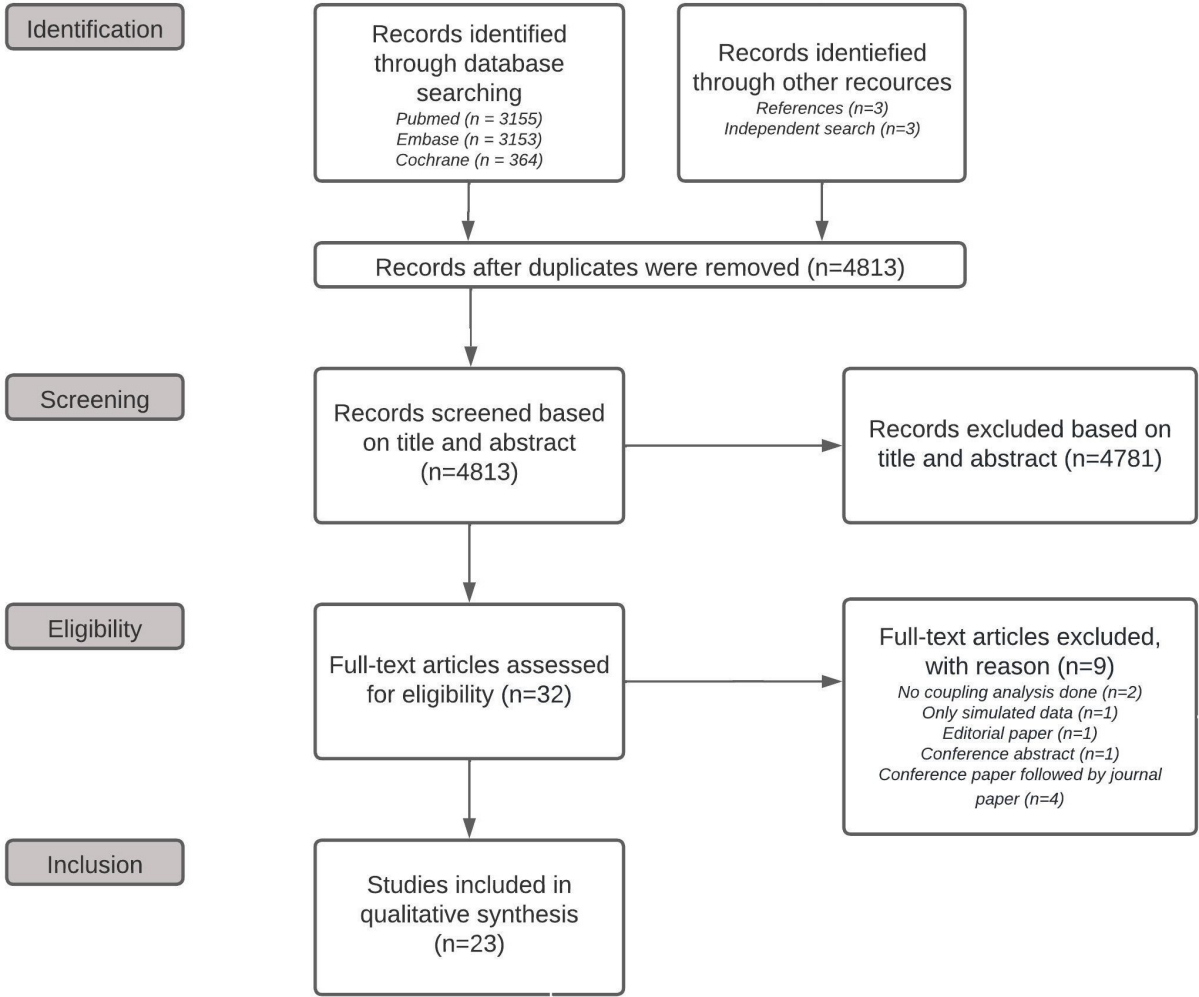
Flowchart of the selection process.

A total of 6,672 studies were identified by searching the indicated databases. An additional six studies were found through other resources; three were found by searching the references of studies included through the database search, while three were found via searches of publications from researchers active in the field of MFCC. The latter three were either conference papers from technically oriented conference proceedings [24] or articles from journals that are not listed in PubMed, Embase, or Cochrane [8, 12]. After removing the duplicate studies, 4,813 unique studies remained, of which 32 were found eligible for full-text screening. After this full-text assessment, 23 studies were included in this review. Fig 1 shows a flowchart of the selection process. The study characteristics and results are summarized in Table 1.

**Table 1:** Study characteristics and results of the included papers.

Author, year	Document type and study design	Population (nr. of recordings)	Gestational age, weeks (nr. of recordings)	In- and exclusion criteria	Data acquisition methods	Coupling assessment method	Results (if coupling ratios are presented, these are M:F)	Presence of cardiac coupling	Direction of coupling	Clinical utility
Hildebrandt, 1979 [9]	Journal article. Longitudinal prospective cohort.	Total 2 (85)	Month 8 or 9 of pregnancy	•*Inclusion*: N/A •*Exclusion*: N/A	•*Method*: fetal and maternal ECG •*Duration*: continuous recording for 3 or 7 nights, respectively; recordings are broken up into 1-hour segments •*Verification of results*: N/A	Synchrograms and phase coherence	• 30/85 (35.3%) recordings with periods of synchronization. •Significant phase preference at 2:1.	Occasional	N/A	N/A
Van Leeuwen, 2003 [26]	Journal article. Longitudinal prospective cohort.	Total 62 (177) • Healthy 35 (139) •FGR 21 (30) •Isolated ectopic beats or short-lived bradycardia 6 (8)	16–42 • 2^nd^ trimester (49) •3^rd^ trimester (128)	•*Inclusion*: N/A •*Exclusion*: persistent arrhythmias	•*Method*: magnetocardiography, •*Duration*: 5-minute recordings. •*Verification of results*: surrogate twin method	Synchrograms and phase coherence	• 164/177 recordings (92.6%) with periods of synchronization. •More synchronization periods in the 3^rd^ trimester than in the 2^nd^ trimester, •Significant phase preference at 3:5 and 4:7. •However, the number and duration of synchronization periods were similar to surrogate data.	Occasional	N/A	N/A
DiPietro, 2004 [7]	Journal article. Longitudinal prospective cohort.	Total 137 (822)	20, 24, 28, 32, 36, 38	•*Inclusion*: non-smoking, uncomplicated singleton pregnancy. •*Exclusion*: preterm delivery, GDM, congenital malformation, fetal death in utero, nonviable delivery, FGR, loss to follow-up.	•*Method*: fetal actocardiography and maternal ECG •*Duration*: 30–50 minutes •*Verification of results*: N/A	Cross-correlation	• No relationship between fetal heart rate and maternal heart rate.	No	N/A	N/A
DiPietro, 2006 [32]	Journal article. Longitudinal prospective cohort.	Total 195 (1170)	20, 24, 28, 32, 36, 38	•*Inclusion*: uncomplicated singleton. •*Exclusion*: preterm delivery, congenital malformations, fetal death in utero, nonviable delivery, condition of antepartum origin detected in the newborn, loss to follow-up.	•*Method*: fetal actocardiograph and maternal ECG •*Duration*: 50 minutes •*Verification of results*: N/A	Cross-correlation	• No relationship between fetal heart rate and maternal heart rate.	No	N/A	N/A
Van Leeuwen, 2009 [27]	Journal article. Prospective cohort.	Total 6 (7)	34–40	•*Inclusion*: N/A •*Exclusion*: N/A	•*Method*: magnetocardigraphy •*Duration*: 40 minutes, which includes 5-minute recordings for 6 different maternal breathing paces (15 cpm, 10 cpm, 20 cpm, 12cpm, spontaneously) •*Verification of results*: surrogate twin method.	Synchrograms and phase coherence	• Synchronization periods in all recordings •Synchronization periods were more prevalent at higher breathing paces •Significant phase preference at 12 cpm: 2:3. •Significant phase preference at 20 cpm: 3:4 and 3:5.	Yes	N/A	Fetal surveillance and the detection of pathological conditions in pregnancy
Riedl, 2009 [36]	Journal article. Retrospective cohort study.	Total 3 (3)	End of pregnancy	•*Inclusion*: N/A •*Exclusion*: N/A	•*Method*: magnetocardigraphy •*Duration*: 5-minute recording at a maternal breathing paces of 20 cpm. •*Verification of results*: surrogate twin method.	Phase locking, Partial Directed Coherence	• Only a few synchronization periods could not be explained by surrogate data •Significant phase preference at 3:5	Occasional	M→F	Detection of prenatal disease or deficit. Assessment of fetal neural integration
Wang, 2013 [29]	Conference paper prospective cohort.	Total 37 (39)	16–40 • 16–26 (10) •27–33 (13) •34–40 (16)	•*Inclusion*: N/A •*Exclusion*: abnormal range of FHR.	•*Method*: abdominal fetal and maternal ECG •*Duration*: 1 minute. •*Verification of results*: N/A	Synchrograms and phase coherence	•Synchronization periods for all recordings •Significant phase preference at 1:2 and 4:5	Yes	N/A	Clinical markers for evaluating antenatal development
Van Leeuwen, 2014 [28]	Journal paper. Retrospective cohort study.	Total 40 (40) •Exercise 21 (21) •Control 19 (19)	36	•*Inclusion*: low-risk pregnancies, singleton, 20–35 years. Subjects in the exercise group exercised for a minimum of 30 minutes, 3 times a week (based on MPAQ questionnaire). •*Exclusion*: excessive artefacts (ectopic beats, preventricular or preatrial contractions)	•*Method*: magnetocardigraphy •*Duration*: 18 minutes •*Verification of results*: surrogate twin method.	Synchrograms and phase coherence	•Synchronization periods in all recordings •Less synchronization in the exercise group •Synchronization is more prevalent at higher breathing paces	Occasional	N/A	Marker for physiological health or development
Khandoker, 2014 [34]	Conference paper. Longitudinal prospective cohort.	Total 45 (66)	•16–25 (22) •26–30 (22) •32–41 (22)	•*Inclusion*: singleton pregnancies •*Exclusion*: N/A	•*Method*: abdominal fetal and maternal ECG •*Duration*: 1 minute •*Verification of results*: N/A	Joint Symbolic Dynamics	•Results indicated significant differences in coupling between early- and mid-gestation as well as early- and late gestation •No differences were seen between mid and late gestation. A variety of coupling patterns can be used to differentiate between gestational groups	Yes	N/A	Clinical markers of healthy prenatal development and fetal cardiac anomalies
Mazbanrad, 2015 [10]	Journal paper Prospective cohort.	Total 65 (65) *The same population as Khandoker 2016*, *but different coupling assessment method*.	16–41 • 16–25 (25) •26–31 (18) •18–41 (22)	•*Inclusion*: normal, singleton pregnancies •*Exclusion*: N/A	•*Method*: abdominal fetal and maternal ECG •*Duration*: 1 minute •*Verification of results*: surrogate twin method.	Transfer Entropy	•Significant TE for 63/65 cases •Significant increase in TE (M→F) and a decreasing trend (F→M) with increasing GA •Decreased delay in TE (M→F)	Yes	Both directions	Assessment of fetal sensory and autonomic nervous system
Khandoker, 2016 [12]	Journal paper. Prospective cohort	Total 66 (66) *The same population as Mazbanrad 2015*, *but different coupling assessment method*.	16–41 • 16–25 (22) •26–31 (22) •18–41 (22)	•*Inclusion*: normal, singleton pregnancies •*Exclusion*: N/A	•*Method*: abdominal fetal and maternal ECG •*Duration*: 1–2 minutes •*Verification of results*: surrogate twin method.	Partial Directed Coherence	•MFCC (M→F) was weak during early gestation, became the strongest in mid-gestation and remained so in late gestation •MFCC (F→M) was the strongest during early gestation and gradually decreased with gestational age progression.	Yes	Both directions	Assessment of fetal well-being
Alangri, 2018 [30]	Conference paper. Prospective cohort	Total 70 (70)Cohort: 44 (44) • Healthy 37 (37) •CHD 7 (7) Added from another database 26 (26)	•<32: healthy (22), CHD (5). •>32: healthy (15), CHD (2). Added from another database (26) •>32 (26)	•*Inclusion*: N/A •*Exclusion*: N/A	•*Method longitudinal cohort*: abdominal fetal and maternal ECG •*Method other database*: Phonocardiography •*Duration*: 1 minute •*Verification of results*: N/A	Synchrogram and phase coherence	•Significant difference in phase coherence index in healthy pregnancies between early GA and late GA •Significant difference in phase coherence index between healthy pregnancies during early GA and fetuses with CHD	Yes	N/A	Marker for development of the autonomic nervous system and impairment of cardiac autonomic activity
Avci, 2018 [11]	Conference paper. Prospective cohort	Total 74 (74)	28–38 • <32 (31) •>31 (43)	•*Inclusion*: low risk pregnant women •*Exclusion*: N/A	•*Method*: magnetocardiography •*Duration*: 6–11 minutes •*Verification of results*: N/A	Transfer Entropy	•TE (M→F) did not significantly change with increasing GA* •TE (F→M) showed a decreasing trend with increasing GA* •	Yes	Both directions	N/A
Khandoker. 2020 [35]	Journal article. Prospective cohort *The same population as Khandoker 2014 and 2019*, *but with different coupling assessment method and abnormal cases are added*.	Total 85 (85) • Healthy 66 (66) Abnormal = fetal bradycardia fetal, tachycardia, premature atrial contraction, different types of CHD 19 (19)	16–41Healthy: • 16–25 (22) •26–30 (22) •32–40 (22) Abnormal •19–38 weeks (19)	•*Inclusion*: N/A •*Exclusion*: N/A	•*Method*: abdominal fetal and maternal ECG •*Duration*: 1 minute •*Verification of results*: N/A	Joint Symbolic Dynamics	Significant differences in the occurrence of a variety of coupling patterns between early and mid/late gestation. Coupling patterns do not capture differences between mid and late gestation Some coupling indices were significantly different for the abnormal group in comparison to the healthy group	Yes	N/A	Marker for healthy prenatal development and fetal cardiac anomalies
Khandoker, 2019 [15]	Journal article. Prospective cohort *Same population as Khandoker 2014 and 2019*, *but different coupling assessment method or abnormal cases are added*.	Total 85 (85) • Healthy 66 (66) •Abnormal = fetal bradycardia fetal, tachycardia, premature atrial contraction, different types of CHD 19 (19)	16–41Healthy: • 16–25 (22) •26–30 (22) •32–40 (22) Abnormal •19–38 weeks (19)	•*Inclusion*: for healthy fetuses as per intrapartum monitoring guidelines (FIGO) •*Exclusion*: N/A	•*Method*: abdominal fetal and maternal ECG •*Duration*: 1 minute •*Verification of results*: surrogate twin method.	Phase locking, Partial Directed Coherence	•Synchronization (M→F)was increased with increasing GA, maximum during mid-gestation •Synchronization (F→M) was decreased with increasing GA •MFCC (F→M) was weaker in abnormal pregnancies and stronger MFCC (M→F) compared to healthy pregnancies	Yes	Both directions	Marker of healthy prenatal development and its deviation; detecting fetal hypoxia
Khandoker, 2020 [14]	Conference paper. Prospective cohort	Total 16 (16)	19–32 weeks	•*Inclusion*: No records of fetal abnormalities •*Exclusion*: N/A	•*Method*: abdominal fetal and maternal ECG •*Duration*: 10 minutes •*Verification of results*: N/A	Phase coherence	•Incorporating coupling parameters improves the estimation of GA compared to using only maternal and fetal HRV features	Yes	N/A	Estimation of fetal gestational age
Khandoker, 2020 [24]	Conference paper. Prospective cohort, animal study	Total 6 mice, 10 fetuses (6)	17.5 days (21 days is full term for mice)^±^	•*Inclusion*: N/A •*Exclusion*: N/A	•*Method*: needle ECG •*Duration measurement*: 15 minutes •*Verification of* results: N/A	Phase coherence	•No significant changes in synchronization during anesthesia	Yes	N/A	N/A
Lobmaier, 2020 [6]	Journal paper. Prospective case-control	Total 104 (104) • Control 53 (53) •Case stressed 51 (51)	>28 weeks • Control 36.7 (53) •Case 36.4 (51)	•*Inclusion*: singleton pregnancies, 18–45 years old, third trimester of pregnancy •*Exclusion*: FGR, fetal malformations, maternal severe illness, maternal drug or alcohol abuse.	•*Method*: abdominal fetal and maternal ECG •*Duration*: 40 minutes •*Verification of results*: N/A	BPRSA	•Fetal stress index was significantly higher in fetuses of stressed mothers when compared to controls.	Yes	M → F	Identification of children at risk for altered neurodevelopmental trajectories due to perinatal stress exposure to allow for early intervention.
DiPietro, 2021 [33]	Journal paper. Prospective cohort.	Total 84 (84)	36.2	•*Inclusion*: obese, singleton, non-smoking, normal pregnancies •*Exclusion*: N/A	•*Method*: Polysomnography and abdominal maternal and fetal ECG •*Duration*: 5 minutes •*Verification of results*: N/A	Cross-correlation	•Synchronization was observed only during WASO (wakefulness after sleep onset)	Occasional	N/A	N/A
Wahbah, 2021 [31]	Journal paper. Prospective cohort	Total 60 (60)	20–41	•*Inclusion*: healthy singleton with no records of fetal abnormalities •*Exclusion*: N/A	•*Method*: abdominal fetal and maternal ECG •*Duration*: 10 minutes •*Verification of results*: N/A	Synchrograms and Phase coherence	•Synchronization changes with GA. •Significant phase preference at 2:3 and 2:4. •Incorporating coupling parameters improves the estimation of GA compared to using only maternal and fetal HRV features.	Yes	N/A	Estimation of fetal gestational age
Tepichin-Castro, 2021 [8]	Journal paper. Longitudinal prospective cohort	Total 22 (44)	•First measurement in third trimester 36.5 (22) •Second measurement during active labour 39.4 (22)	•*Inclusion*: low-risk pregnant women •*Exclusion*: N/A	•*Method*: abdominal fetal and maternal ECG •*Duration*: 5 minutes •*Verification of results*: N/A	Joint Symbolic Dynamics	•Stronger coupling indices during active labour as compared to third trimester	Yes	N/A	Monitoring during labour to assess fetal well-being of both mother and fetus.
Alkhodori, 2022 [23]	Journal article. Prospective cohort (local dataset for testing and training AI model) and retrospective cohort (Physionet dataset for validation)	Total 114 (941) Local dataset: 109 (873) Physionet dataset: 5 (68)	•Local dataset: 20–40 (873) •Physionet dataset: 38–41 weeks (68)	•*Inclusion*: healthy fetal cardiac condition •*Exclusion *: maternal cardiovascular condition	•*Method*: fetal and maternal ECG, •*Duration*: 1 minute. •*Verification of results*: results from deep learning are compared to phase coherence index results (considered as the group truth)	Synchrograms and phase coherence index (as ground truth) Deep learning (termed deep coherence)	•The number of recordings with coupling is not specified. •Significant phase preference at 1:2, 2:3, and 3:5. •Deep coherence was 90% accurate in identifying the phase of coupling (AUROC > 0.93) •Phase preferences change with GA •Phase preferences are significantly associated with maternal BMI and age.	Yes	N/A	Continuous monitoring of fetal condition to improve triaging using lower-cost devices with less side-effect than those currently used.
Khandoker, 2022 [40]	Journal paper Case-controlled animal study	Total 27 mice (27), 48 fetuses (48) - Atropine injection 9 mice (9), 14 fetuses (14) - Propranolol injection 9 mice (9), 17 fetuses (17) - Saline injection 9 mice (9), 17 fetuses (17)	•17.5 days (21 days is full term for mice)	•*Inclusion*: N/A •*Exclusion*: N/A	•*Method*: needle ECG •*Duration*: 20 minutes (injection after 10 minutes) •*Verification of results*: saline injection •	Phase coherence	•Atropine injection increases ratio 1:4 and decreases ratios 1:2 and 1:3. •Atropine injection increases ratio 1:4 and 1:5, as well as decreases ratio 1:2. •Coupling ratios are not significantly affected by saline injection.	Yes	N/A	Understanding the role of maternal autonomic activity in fetal development and complications.

**BPRSA**: bivariate phase rectified signal averaging, **CHD**: congenital heart disease, **CPM**: cycles per minute, **F**→**M**: Fetal to Maternal Direction, **FGR**: Fetal growth restriction, **GA**: gestational age, **GDM**: Gestational diabetes mellitus, **HRV**: Heart Rate Variability, **M**→**F**: Maternal to Fetal Direction, **MFCC**: Maternal-Fetal Cardiac Coupling, **MPAQ**: Modifiable Physical Activity Questionnaire, **TE**: Transfer Entropy. P-values of 0.05 were used to indicate significance for all articles included in this review. ^*^P-value of 0.01 (for all other analysis, a P-value of 0.05 was used to demonstrate significance). ^±^Note that contrary to humans, maternal HR in mice is lower than fetal HR.

Hereafter we will elaborate on four aspects of the results reported in the table, namely: the different methodologies that have been used to capture MFCC; the results on the existence and direction of MFCC; the physiological explanations offered for MFCC; and the potential clinical possibilities of MFCC suggested in the included studies.

### 3.1 MFCC: Methodologies

Broadly, MFCC analyses may be assigned to three groups: synchronization or coordination, describing a fixed relationship between two signals in either phase or time; pattern-matching, where the aim is to see if similar activity occurs in both signals; and modulation, which implies that changes in one signal results in or relates to changes in another [25]. The methodology of earlier studies investigating MFCC focused on finding periods of synchronization with synchrograms and corresponding phase coherence indices [9, 26–31], as well as corresponding patterns between maternal and fetal cardiac activity with cross-correlation [7, 32, 33]. In line with the latter, joint-symbolic dynamics was subsequently used to investigate whether maternal and fetal HR behavior corresponded to each other [8, 34, 35]. In more recent studies, the focus mostly shifted towards methods more closely associated with modulation [6, 10–12, 36]. A summary of these methods is presented here.

#### 3.1.1 Synchrograms and phase coherence index

Synchrograms are a visual representation of the relative phases of the maternal and fetal heartbeats. The more fixed the relationship between the relative phases of the maternal and fetal heartbeats are, the higher the coherence is between them. When periods of sufficient coherence occur (i.e., where the metric describing coherence exceeds a prespecified threshold), it is determined that phase locking occurs in this period of the signal. The expected ratio between the heartbeats needs to be defined a-priori. Periods where phase locking is detected are reported either as the number of occurrences of these phase locking periods or as their prevalence in the signals (e.g., phase synchronization of two fetal heartbeats to one maternal heartbeat, 2:1, was found in 8% of the signal). Such analyses do not address the potential directionality of MFCC. In other words, it does not say whether the fetal HR affects the maternal HR or the other way around. Additionally, the final study included in Table 1 uses an artificial intelligence method known as *Deep coherence* [37]. This method is a deep learning implementation of the phase coherence index, where the deep learning model seeks to identify phases of synchronization in correspondence with what would be found with the original method described above, but without any mathematical derivations, pre-processing steps, or signal transformations to the input data [37].

#### 3.1.2 Cross-correlation

This method assesses the similarity of two signals as a function of the displacement of one signal relative to the other. Therefore, cross-correlation accounts for a possible lag in the relationship between the maternal and fetal heartbeats. Therefore, it is possible to see when a pattern in one signal precedes the pattern in the other which may offer some indication of the directionality of the coupling. A higher cross-correlation value, therefore, implies stronger coupling.

#### 3.1.3 Joint-symbolic dynamics (JSD)

JSD is a processing technique where information in a complex signal is simplified by replacing it with symbols (known as course-graining). In the case of MFCC, for example, each heartbeat may be replaced with a symbol indicating that it is increased (I), constant (C), or decreased (D) to the previous beat. In this way, patterns are detected in the signal, for example, DDD would indicate a sustained decrease in HR. In JSD, both the maternal and fetal HR signals are replaced with such symbols. Thereafter, the overlap between the two signals is measured with for example cross-correlation methods or cross-sample entropy.

#### 3.1.4 Transfer entropy (TE)

TE assesses whether having information about the past activity of signal 1 reduces the information needed to describe the current or future activity in signal 2. The more the past information of signal 1 reduces the uncertainty in describing signal 2, the higher the information flow, and therefore TE, is from signal 1 to signal 2. A higher TE value suggests stronger coupling. TE inherently assumes a direction between the interactions.

#### 3.1.5 Granger causality and partial directed coherence (PDC)

Granger causality operates on a similar principle to TE. If past information from signal 1 is useful in *predicting* the current state of signal 2, signal 1 is said to cause signal 2. Granger causality therefore inherently presumes a directionality between the information flow of the two signals. PDC, which is said to determine the intensity of information flow, is based on the principle of Granger causality. However, while Granger causality is assessed in the time domain, PDC is calculated using the frequency information of the time series. A higher causality or coherence value indicates stronger coupling.

#### 3.1.6 Bivariate phase rectified signal averaging (BPRSA)

BPRSA assumes that changes in signal 1 (the trigger signal) result in or correspond to changes in signal 2 (the target signal). Anchor points–which are defined as the location of certain signal phases of interest, such as where the HR decelerates–are identified on the trigger signal. A signal segment is isolated around each anchor point which is sufficiently long to capture the expected interactions. All identified signal segments are then aligned and averaged. This process is then repeated in the target signal, using the anchor points identified in the trigger signal. If no relationship exists between the two signals, then this averaging should result in a flatline signal. However, if a relationship indeed exists, there should be an observable response in both averaged-out signals, implying that activity in the trigger signal is in some way influencing the target signal. By specifying the trigger and target signal, a directional relationship is inherently being investigated, yet, changes in both trigger and target signals may be modulated by a tertiary mechanism. Subsequently, an observed relationship does not imply causality.

#### 3.1.7 Methods used in papers included in this scoping review

Synchrograms and phase coherence index were used to investigate MFCC in 11 (47.9%) of the included studies, cross-correlation was used in three (13.0%) studies, JSD was investigated in three (13.0%) studies, TE in two (8.7%) studies, Granger causality and PDC in three studies (13.0%), and finally BPRSA was used to investigate MFCC in one study (4.4%).

### 3.2 MFCC: Presence and directionality

Of the included studies, 21 (91.3%) found that MFCC existed, at least, occasionally. The remaining two (8.3%) studies, which used cross-correlation to capture MFCC [7, 32], did not find any evidence of MFCC.

Studies investigating the phase locking between the maternal and fetal cardiac systems using synchrograms and phase coherence indices found occasional periods of synchronization. Using these methodologies, researchers demonstrated how the prevalence of these periods of synchronization increases or decreases under certain conditions such as different maternal respiration rates [27, 28], progressing GA [26, 30], or regular maternal exercise [28]. While epochs of synchronization were present in all recordings regardless of the maternal respiration rate, synchronization was more prevalent at higher rates of respiration. On the other hand, mothers who exercised regularly during pregnancy had lower incidences of MFCC than their less active controls [28]. GA seems to influence the synchronization ratio as it gradually reduces with progressing GA. However, another study could not demonstrate the influence of progressing GA on synchronization. Two studies using cross-correlation did not find MFCC. A third performed their analysis using nighttime recordings–owing to the reduced effect of motion artifacts and external stimuli during this period–and stratified their cross-correlation analysis by sleep stages. This study reported occasional MFCC in the period of wakefulness after sleep onset period [7, 32, 33].

The method of JSD was used in three studies, in each of which MFCC was captured and found to change with progressing GA. MFCC patterns were significantly different between the early- and mid-GA groups as well as between the early- and late-GA groups (16–25 weeks, 26–31 weeks, and 32–41 weeks GA, respectively) [34]. Furthermore, one of these studies compared MFCC in women between their third trimester and during labor, finding stronger MFCC patterns during labor as compared to the third trimester [8]. The third study found altered MFCC patterns in fetuses with cardiac abnormalities in comparison to healthy fetuses. The changes in the MFCC patterns of the complicated pregnancies compared to the healthy ones differed depending on the type of fetal cardiac anomaly [35].

Furthermore, MFCC was investigated with TE, and researchers found MFCC in both directions. We introduce the terminology of MFCC_M→F_ if information flows from the mother to the fetus and MFCC_F→M_ for the alternative. The mentioned directionality should not be interpreted as implying causality. MFCC_M→F_ was found to either stay constant or increase only slightly with progressing GA, while MFCC_F→M_ was found to reduce with advancing gestation [10, 11]. Studies using PDC or Granger causality similarly found MFCC to be present in both directions with the strength of MFCC_M→F_ increasing with GA while the strength of MFCC_F→M_ decreased with progressing GA [12, 15, 36].

### 3.3 MFCC: Physiological pathways

No studies included in this review described specific investigations into the physiological pathways that are responsible for MFCC. However, some researchers suggest that the maternal heart rhythm mechanically or vibroacoustically stimulates the fetal heart [26–28]. The pulsation of the maternal arteries causes vibrations which may be sensed or heard by the fetus. When the frequency of these vibrations approaches that of the fetal heart rhythm, the fetal heartbeat may become entrained to the maternal heart [9, 38, 39]. Furthermore, researchers suspect that the autonomic nervous system (ANS) serves as a pathway for MFCC. Consequently, small scale studies performed on mice models were used to test this hypothesis. These studies revealed alterations in MFCC under maternal sympathetic or para-sympathetic blockade [40], although no clear conclusions could be drawn as to the role of the ANS in MFCC.

### 3.4 MFCC: Clinical relevance

Overall, researchers suggest that assessing MFCC may serve as a tool to assess fetal well-being during pregnancy and labor, and to track fetal development. However, three clinical applications of MFCC have been specifically investigated: first, the potential for using MFCC indices to discriminate between normal and abnormal fetuses [15, 30, 35]; second, estimating GA based on MFCC indices in rural or remote setups where ultrasound technology or expertise is not available [14, 31]; and third, using MFCC as an index of prenatal exposure to maternal stress [6].

Three studies specifically investigated abnormal fetuses in comparison to healthy fetuses. Two of these studies investigated a heterogeneous group of pregnancies with fetal cardiac anomalies or fetal cardiac heart rhythm disorders such as fetal bradycardia, fetal tachycardia, or premature atrial contractions. The first study, using JSD, found stronger MFCC patterns for the abnormal cases when compared to pregnancies with healthy fetuses [35]. The second study, using PDC, found decreased MFCC_F→M_, while MFCC_M→F_ was increased compared to healthy fetuses [15]. Finally, one other study using phase locking found significant differences in phase coherence indices between fetuses affected by different types of congenital heart diseases and healthy fetuses [30].

Two other studies showed that incorporating synchronization and phase coherence parameters could improve the estimation of GA with regression models when compared against models using only maternal and fetal HR variability features. When compared against the gold standard of establishing GA from crown-rump length, the best performing model had a mean root mean square error of 2.67 weeks [14, 31].

Finally, one study used BPRSA to investigate MFCC in fetuses with stressed mothers (as assessed with the Perceived Stress Scale index) [6]. Features from the BPRSA analysis were used to develop a fetal stress index (FSI). The FSI was significantly higher in fetuses with stressed mothers compared to controls.

## 4. Discussion

Although there is heterogeneity in methodologies used and populations assessed in the studies included in this scoping review, it seems that MFCC does indeed exist, both from the mother to her fetus (MFCC_M→F_) as well as from the fetus to its mother (MFCC_F→M_). Furthermore, there is potential clinical value in assessing MFCC for monitoring fetal well-being and tracking fetal development.

While analyses using cross-correlation did not yield convincing evidence for MFCC [7, 32], phase synchronization, along with its phase coherence index, captured occasional MFCC between the maternal-fetal pair [9, 14, 15, 26–31, 36, 40]. Researchers have also remarked that using cross-correlation to investigate associations between timeseries data (such as maternal and fetal heart rhythms) often leads to an underestimation of the strength of the association [7]. Considering this limitation and further considering that most studies support the existence of MFCC, we conclude that cross-correlation is not an appropriate method for capturing MFCC.

The seemingly intermittent nature of MFCC motivated investigations into the conditions under which MFCC occurs. While regular maternal physical exercise resulted in less synchronization between maternal and fetal HR [28], higher instances of MFCC were found at higher maternal respiration rates [27]. Furthermore, MFCC also varies with progressing pregnancy both in strength and direction. In early pregnancy, the influence is mainly from the fetus to the mother, while in later pregnancy MFCC_M→F_ is dominant [11, 12, 15, 41].

The etiology of MFCC is currently unknown. Some researchers suggest that this type of coupling may be mechanically or acoustically driven [26–28]. Similar to cardiac rhythms becoming entrained to locomotor actions in cardiac-locomotor coupling (i.e., when the frequency of a rhythmic activity such as walking becomes close to the frequency of the HR, or a fixed factor thereof, and the two synchronize to each other) [42], the fetal heart rhythm may become entrained to the forcing maternal cardiac oscillator, i.e., the maternal pulse waves [9]. Furthermore, the fetal HR changes in reaction to the mechanical energy from the maternal vessels may be enhanced by the fetus’s auditory perception of the frequency range of the pulsating maternal arteries–a phenomenon called the vibroacoustic effect [26–28]. This could explain the increasing strength of MFCC_M→F_ with GA, as the fetal auditory system is only fully developed at 27 weeks of gestation [43]. The vibroacoustic effect has been observed in adults where a frequency-lock was found in reaction to an external acoustic signal, but only when the frequency was similar to that of the subject’s HR [38]. The same might be happening to the fetal HR in the case of MFCC, although this would rarely be observed as the HR frequencies of the mother and fetus would be too far apart to induce MFCC under normal circumstances.

Subsequently, it stands to reason that a higher incidence of MFCC may be observed at higher maternal HR. This aligns with findings suggesting more periods of MFCC during quicker maternal respiration; increased respiration narrows the maternal interval between successive heartbeats, potentially encouraging the entrainment of the fetal rhythm to that of its mother [27]. Similarly, MFCC was less common in pregnancies where mothers had higher cardiovascular fitness and correspondingly lower resting respiratory rate and HR [28]. However, rather than changes in maternal cardiac rhythm modulating changes in the fetal rhythm (or vice versa), it may be possible that a third system is driving changes in both these systems [33]. Specifically, it is feasible that maternal respiration acts as a common driving force, simultaneously modulating both the maternal and the fetal HR [27]. This modulating effect of maternal respiration is yet to be directly investigated. However, a faster-paced maternal respiratory rate did induce higher instances of MFCC [27]. We propose that the movement of the maternal diaphragm may also play a role here, exhorting a vibroacoustic effect on both the maternal and fetal cardiac system, but this has not been investigated.

The increased strength of MFCC_M→F_ with progressing GA is likely linked to the maturation of the fetal ANS, reaching maturity around the transition from the second to the third trimester [44]. With gestational progression, the increasingly stable and finely tuned fetal ANS may lead to an enhanced fetal cardiac reaction to maternal input [10, 41, 45]. On the other hand, while respiratory sinus arrhythmia is typically present in the mother, in the fetus it is present in increasing strength from 32 weeks GA onwards [44]. Theoretically, the fetal HR would become more closely coupled with its own respiratory system from this point onward. Yet, this manifests as a decrease in MFCC_F→M_ with progressing GA rather than a decrease in MFCC_M→F_, further highlighting the complexity and dearth of knowledge concerning MFCC.

Additionally, the adrenergic innervation of the uterine wall may play an important role in MFCC_F→M_ [46, 47]. Fetal movements may stimulate the maternal sympathetic nervous system, resulting in higher maternal HR. Theoretically, this effect would increase with gestational progression as larger fetuses are capable of stronger movements. However, the opposite is observed; MFCC_F→M_ decreases with progressing pregnancy. This decrease is likely due to the maternal ANS becoming increasingly hypo-responsive to external stimuli, such as fetal movements, during healthily progressing pregnancy [41, 48, 49]. Small-scale studies using mice models also support the hypothesis of the ANS playing a central role in MFCC as indices of MFCC in pregnant mice reveal an antagonistic response to maternal sympathetic or para-sympathetic blockade [40]. However, these animal studies used phase coherence for the assessment of MFCC and were therefore not able to assess directionality.

Still, even though the etiology of MFCC is not yet clear, results do suggest that assessments of this coupling may have clinical relevance. Indices of MFCC are altered in pregnancies with fetuses affected by cardiac arrhythmias or fetal cardiac anomalies compared to healthy fetuses [15, 30, 35]. Furthermore, MFCC parameters have been used to estimate fetal GA fairly successfully against the gold standard [14, 31].

Maternal stress during pregnancy has also been found to affect MFCC [6]. Based on this finding, researchers have developed an FSI (based on MFCC features) to identify infants at risk for altered neurodevelopmental trajectories due to perinatal stress exposure [6]. While no further clinical applications have been investigated, the most common suggestion for clinical applications is tracking fetal neurodevelopment to screen for abnormalities.

The effect of maternal complications on MFCC has yet to be explored. Such analyses are potentially interesting since, as previously discussed, MFCC seems to be affected by autonomic changes, and complications such as hypertensive disorders of pregnancy are associated with dysfunctional autonomic regulation [50]. Furthermore, assessments of maternal-infant cardiac coupling in the immediate postnatal period–preferably in preterm infants where the autonomic behavior is still similar to that of the fetus–may be illuminating. In such a study design, various possible influencing factors could be examined under controlled conditions, for example, changes in maternal respiration rate or HR. Additionally, synchronization under specific (patho)physiological conditions such as fetal behavioral state or fetal hypoxemia should be investigated. The latter might be particularly interesting. While the evolutionary driver behind MFCC is unknown, it may be in some way related to the oxygenation of the fetus; i.e., lower oxygenation levels in the fetal blood could trigger increases in maternal HR to increase gas exchange via the placenta [51]. On the other hand, when maternal oxygen levels decrease, the fetal HR responds by increasing the fetal HR [52].

Several limitations exist that affect the investigation of MFCC. While each of the studies in this scoping review is limited in some ways, there are also inherent difficulties in studying MFCC. First, since time-synchronized maternal and fetal HR are needed, options for measurement technologies to capture MFCC are limited. While magnetocardiography can be used, it is impractical, owing to the size and expense of the equipment, therefore leaving abdominal electrocardiophysiology (ECG) as the pragmatic option. Fetal HR can be difficult to accurately detect from abdominal ECG and signals capturing the electrophysiological activity of the fetal heart are typically weak (i.e., of low signal-to-noise ratio). However, recent advancements in the field of fetal electrocardiography have greatly contributed to solving this problem by providing higher quality fetal signals that enable more accurate MFCC investigation [53]. Second, the majority of methods used to assess the coupling between systems derive from different scientific domains and are not specifically designed to study coupling between physiological systems, which might make them less effective. Third, the studies included in this review reveal that there is no consensus on the definition of MFCC. This is important since the definition of coupling determines the method by which researchers chose to study its potential occurrence; a presumption of fixed phase ratios between the maternal and fetal heartbeats would most likely lead to analysis via synchrograms, while hypothesizing that modulations in one signal lead to or correspond to changes in the other would likely result in a TE or BPRSA analysis.

Lastly, a deep learning approach called deep coherence was recently proposed in the field of MFCC research [37]. Deep learning methods like deep coherence may help to reduce the need for a priori assumptions and processing. However, from this review it is clear that while MFCC does seem to exist, our understanding of MFCC is limited. Therefore, techniques which are not fully explainable to capture MFCC should be used with caution. Rather, it may be beneficial for future research to first directly compare known coupling techniques for the assessment of MFCC to narrow down those which are useful [54]. Furthermore, more research is necessary to probe the pathway behind and nature of MFCC.

## 5. Conclusion

We conclude that the studies included in this scoping review suggest that MFCC does exist and that its strength and direction change with progressing GA. Although the physiological pathways of MFCC are not yet sufficiently substantiated, assessing MFCC during pregnancy may offer opportunities to assess fetal development and well-being and may potentially aid in detecting fetal (cardiac) abnormalities.

## Supporting information

S1 Checklist PRISMA-ScR checklist. (DOCX)

S1 File Search strategy (Pubmed, Embase). (DOCX)

## Abbreviations

ANS: Autonomic nervous system
BPRSA: Bivariate Phase Rectified Signal Averaging
FSI: Fetal Stress Index
F→M: Fetus to mother
GA: Gestational age
HR: Heart rate
JSD: Joint-Symbolic Dynamics
MFCC: Maternal Fetal Cardiac Coupling
M→F: Mother to fetus
PDC: Partial Directed Coherence
TE: Transfer Entropy
