# Supplementary material for: Evidence and clinical relevance of maternal-fetal cardiac coupling: A scoping review
Source: PLoS One. 2023 Jul 12;18(7):e0287245. doi: 10.1371/journal.pone.0287245 (PMC10337870; doi:10.1371/journal.pone.0287245)
Supplement: S1 File — (DOCX) [file pone.0287245.s002.docx]

Pubmed:

"Maternal-Fetal Relations"[Mesh] OR "Mother-Child Relations"[Majr] OR "maternal fetal relation*"[tiab] OR "mother-fetus"[tiab] OR "mother fetus"[tiab] OR "mother fetus relation*"[tiab] OR "Fetal Heart"[Mesh] OR "fetal heart rate*"[tiab] OR "fetus heart rate*"[tiab] OR "foetal heart rate*"[tiab] OR "heart rate synchronization" [tiab] OR "maternal fetal heart rate"[tiab] OR "maternal-fetal"[tiab] OR "fetal-maternal"[tiab] OR "fetal-maternal heart"[tiab] OR "fetal-maternal exchange"[tiab] OR "fetal circulatory"[tiab] OR "mother-child*"[tiab]

AND

"Pulse"[Mesh] OR "cardiac coupling"[tiab] OR coupling*[tiab] OR "coordination"[tiab] OR regulation*[tiab] OR synchronization[tiab] OR synchronisation[tiab] OR rhythm*[tiab] OR pulse*[tiab] OR (cardio*[tiab] AND coupling[tiab]) OR cardial[tiab] OR "cardiorespiratory coupling"[tiab] OR "cardiorespiratory synchronization"[tiab] OR "cardiorespiratory coordination"[tiab]

Embase:

'fetus heart rate'/exp OR 'fetal heart rate':ti,ab,kw OR 'fetus heart beat':ti,ab,kw OR 'fetus heart rate':ti,ab,kw OR 'foetal heart rate':ti,ab,kw OR 'foetus heart rate':ti,ab,kw OR 'mother child relation'/exp OR 'child mother relation':ti,ab,kw OR 'maternal role':ti,ab,kw OR 'mother child interaction':ti,ab,kw OR 'mother child relation':ti,ab,kw OR 'mother child relationship':ti,ab,kw OR 'mother infant relation':ti,ab,kw OR 'fetus circulation'/exp OR 'fetal circulation' OR 'fetal placenta circulation' OR 'feto placental circulation' OR 'fetoplacental circulation' OR 'fetus circulation' OR 'fetus circulation placenta' OR 'fetus placenta circulation' OR 'foetal circulation' OR 'foetal circulatioplacenta' OR 'foetal placenta circulation' OR 'foetus circulation' OR 'foetus placenta circulation' OR 'placenta circulation fetus' OR 'maternal fetal':ti,ab,kw OR 'fetal maternal':ti,ab,kw OR 'maternal-fetal':ti,ab,kw OR 'fetal-maternal':ti,ab,kw

AND

'pulse rate'/exp OR 'arterial pulse rate':ti,ab,kw OR 'artery pulse rate':ti,ab,kw OR 'pulse':ti,ab,kw OR 'pulse frequency':ti,ab,kw OR 'pulse rate':ti,ab,kw OR 'coupling'/exp OR 'coupling':ti,ab,kw OR 'cardial':ti,ab,kw OR 'regulation':ti,ab,kw OR 'cardiorespiratory coupling' OR 'cardiorespiratory synchronization':ti,ab,kw OR 'cardiorespiratory coordination':ti,ab,kw
