# Supplementary material for: Evidence and clinical relevance of maternal-fetal cardiac coupling: A scoping review
Source: PLoS One. 2023 Jul 12;18(7):e0287245. doi: 10.1371/journal.pone.0287245 (PMC10337870; doi:10.1371/journal.pone.0287245)
Supplement: Peer review history

# Peer review: Decision Letter 0

**DOI:** 10.1371/journal.pone.0287245.r001

**Authors:** Ahmed Mohamed Maged

5 Apr 2023

PONE-D-22-34923Evidence and clinical relevance of maternal-fetal cardiac coupling: a scoping review.PLOS ONE

Dear Dr. Nichting,

Thank you for submitting your manuscript to PLOS ONE. After careful consideration, we feel that it has merit but does not fully meet PLOS ONE’s publication criteria as it currently stands. Therefore, we invite you to submit a revised version of the manuscript that addresses the points raised during the review process.

==============================

Please respond to all reviewers comments one by one

==============================

Please submit your revised manuscript by May 20 2023 11:59PM. If you will need more time than this to complete your revisions, please reply to this message or contact the journal office at plosone@plos.org. When you're ready to submit your revision, log on to https://www.editorialmanager.com/pone/ and select the 'Submissions Needing Revision' folder to locate your manuscript file.

Please include the following items when submitting your revised manuscript:

- A rebuttal letter that responds to each point raised by the academic editor and reviewer(s). You should upload this letter as a separate file labeled 'Response to Reviewers'.
- A marked-up copy of your manuscript that highlights changes made to the original version. You should upload this as a separate file labeled 'Revised Manuscript with Track Changes'.
- An unmarked version of your revised paper without tracked changes. You should upload this as a separate file labeled 'Manuscript'.

If you would like to make changes to your financial disclosure, please include your updated statement in your cover letter. Guidelines for resubmitting your figure files are available below the reviewer comments at the end of this letter.

If applicable, we recommend that you deposit your laboratory protocols in protocols.io to enhance the reproducibility of your results. Protocols.io assigns your protocol its own identifier (DOI) so that it can be cited independently in the future. For instructions see: https://journals.plos.org/plosone/s/submission-guidelines#loc-laboratory-protocols. Additionally, PLOS ONE offers an option for publishing peer-reviewed Lab Protocol articles, which describe protocols hosted on protocols.io. Read more information on sharing protocols at https://plos.org/protocols?utm_medium=editorial-email&utm_source=authorletters&utm_campaign=protocols.

We look forward to receiving your revised manuscript.

Kind regards,

Ahmed Mohamed Maged, MD

Academic Editor

PLOS ONE

Journal Requirements:

When submitting your revision, we need you to address these additional requirements.

1. Please ensure that your manuscript meets PLOS ONE's style requirements, including those for file naming. The PLOS ONE style templates can be found at 

https://journals.plos.org/plosone/s/file?id=wjVg/PLOSOne_formatting_sample_main_body.pdf and 

https://journals.plos.org/plosone/s/file?id=ba62/PLOSOne_formatting_sample_title_authors_affiliations.pdf

2. Please include captions for your Supporting Information files at the end of your manuscript, and update any in-text citations to match accordingly. Please see our Supporting Information guidelines for more information: http://journals.plos.org/plosone/s/supporting-information.

3. Please review your reference list to ensure that it is complete and correct. If you have cited papers that have been retracted, please include the rationale for doing so in the manuscript text, or remove these references and replace them with relevant current references. Any changes to the reference list should be mentioned in the rebuttal letter that accompanies your revised manuscript. If you need to cite a retracted article, indicate the article’s retracted status in the References list and also include a citation and full reference for the retraction notice.

Additional Editor Comments:

Please respond to all reviewers' comments one by one

[Note: HTML markup is below. Please do not edit.]

Reviewers' comments:

Reviewer's Responses to Questions

**Comments to the Author**

1. Is the manuscript technically sound, and do the data support the conclusions?

The manuscript must describe a technically sound piece of scientific research with data that supports the conclusions. Experiments must have been conducted rigorously, with appropriate controls, replication, and sample sizes. The conclusions must be drawn appropriately based on the data presented. 

Reviewer #1: No

Reviewer #2: Yes

Reviewer #3: Partly

Reviewer #4: Yes

Reviewer #5: Yes

**********

2. Has the statistical analysis been performed appropriately and rigorously? 

Reviewer #1: No

Reviewer #2: N/A

Reviewer #3: N/A

Reviewer #4: N/A

Reviewer #5: N/A

**********

3. Have the authors made all data underlying the findings in their manuscript fully available?

The PLOS Data policy requires authors to make all data underlying the findings described in their manuscript fully available without restriction, with rare exception (please refer to the Data Availability Statement in the manuscript PDF file). The data should be provided as part of the manuscript or its supporting information, or deposited to a public repository. For example, in addition to summary statistics, the data points behind means, medians and variance measures should be available. If there are restrictions on publicly sharing data—e.g. participant privacy or use of data from a third party—those must be specified.

Reviewer #1: No

Reviewer #2: Yes

Reviewer #3: Yes

Reviewer #4: Yes

Reviewer #5: Yes

**********

4. Is the manuscript presented in an intelligible fashion and written in standard English?

PLOS ONE does not copyedit accepted manuscripts, so the language in submitted articles must be clear, correct, and unambiguous. Any typographical or grammatical errors should be corrected at revision, so please note any specific errors here.

Reviewer #1: No

Reviewer #2: Yes

Reviewer #3: Yes

Reviewer #4: Yes

Reviewer #5: Yes

**********

5. Review Comments to the Author

Please use the space provided to explain your answers to the questions above. You may also include additional comments for the author, including concerns about dual publication, research ethics, or publication ethics. (Please upload your review as an attachment if it exceeds 20,000 characters)

Reviewer #1: Submitted article:- "Evidence and clinical relevance of maternal-fetal cardiac coupling: a scoping review" is out of scope and it not technically sounded. writting formate is not valid and given figure 1 is not in good quality.

Reviewer #2: Thomas et al. presented a fascinating scoping review on maternal-fetal cardiac coupling using available literature available on biomedical literature search engines. As a basic medicine researcher, I think it would be worth publishing such an article in PlosOne because it is well prepared and the findings are exciting. The introduction and literature search method to search literature is thorough. However, several inconsistencies throughout the article have been noticed. Please find below a couple of comments and concerns that the authors should address-

1. In the abstract section line 50, it would be clearer if the author added some words saying they included all the articles available regardless of the publishing years.

2. On page 7 (Line 120), using a regular citation format to register the review protocol would be better than the current DOI link.

3. On page 7 (Line 129), what criteria have been searched of a researcher to consider them to call them active researchers in the field of MFCC? It should be mentioned to avoid the biases of literature searches.

4. On page 8 (Line 153), how were the duplicates checked? It should be clearly mentioned.

5. On page 14 (Line 265), avoiding words like “Please note that” would be better. The author needs to revise it.

6. On page 14 (Line 353), what is the basis of the proposed hypothesis? Is it based on some logic or some reference articles? It could be mentioned here.

7. The article title should be in lowercase on page 27 (Line 551) in reference 37.

8. Lastly, I recommend the authors to use a reference management software package like EndNote or Mendeley if they did not use it for this manuscript.

Reviewer #3: This is an important scoping review addressing the phenomenon of maternal-fetal cardiac coupling (MFCC) and its potential clinical implications. The review did not explain how the difference between the normal fetal heart rate (120 – 160 bpm) and the normal mother's heart rate (60 – 100 bpm) could influence maternal-fetal cardiac coupling. Specifically, the authors mentioned that synchronization was more prevalent at higher respiration rates, while mothers who exercised regularly had lower incidences of MFCC than their less-active controls. High respiration and regular exercise are associated with a faster heart rate, which could hypothetically result in a higher incidence of MFCC. However, the outcomes from these two situations are entirely the opposite, which raises the question of the effect of the difference between the mother and fetal heart rates.

Reviewer #4: The manuscript reads well and provides detailed scoping review on the topic of MFCC. Further, it directs future studies on mechanisms and nature of MFCC that may have implications on determining fetal cardiac health. I think it fulfils the need of scoping reviews.

Reviewer #5: Dear author i have gone through your scoping review, i must say that a very nicely literature is searched and written that the reader could understand the background of the study. A clear discussion written

**********

6. PLOS authors have the option to publish the peer review history of their article (what does this mean?). If published, this will include your full peer review and any attached files.

If you choose “no”, your identity will remain anonymous but your review may still be made public.

**Do you want your identity to be public for this peer review?** For information about this choice, including consent withdrawal, please see our Privacy Policy.

Reviewer #1: No

Reviewer #2: **Yes: **A.S.M. WALIULLAH

Reviewer #3: No

Reviewer #4: No

Reviewer #5: **Yes: **Aisha Wali

**********

[NOTE: If reviewer comments were submitted as an attachment file, they will be attached to this email and accessible via the submission site. Please log into your account, locate the manuscript record, and check for the action link "View Attachments". If this link does not appear, there are no attachment files.]

While revising your submission, please upload your figure files to the Preflight Analysis and Conversion Engine (PACE) digital diagnostic tool, https://pacev2.apexcovantage.com/. PACE helps ensure that figures meet PLOS requirements. To use PACE, you must first register as a user. Registration is free. Then, login and navigate to the UPLOAD tab, where you will find detailed instructions on how to use the tool. If you encounter any issues or have any questions when using PACE, please email PLOS at figures@plos.org. Please note that Supporting Information files do not need this step.

# Author comment: Author response to Decision Letter 0

**DOI:** 10.1371/journal.pone.0287245.r002

18 May 2023

Máxima MC, Veldhoven

Department of Gynaecology and Obstetrics

P.O. box 7777, 5500MB Veldhoven, The Netherlands

18 May 2023

Veldhoven, The Netherlands

Subject: Rebuttal letter revisions PONE-D-22-34923

Dear Prof. Ahmed Mohamed Maged,

We hereby submit the revised version of the manuscript, duly addressing the points brought forth during the review process for the article entitled "Evidence and clinical relevance of maternal-fetal cardiac coupling: a scoping review". Please find a pointwise response to the comments from the reviewers below. Corresponding changes in the manuscript are highlighted in yellow. 

We hope that this letter sufficiently addresses all comments. If we can provide you with any further information, do not hesitate to reach us.

Yours sincerely, 

On behalf of all authors,

Drs. T.J. Nichting

Corresponding author

Mail: thomas.nichting@mmc.nl

Phone: +31 64 864 9152

Reviewers’ comments: 

Reviewer 1: "Evidence and clinical relevance of maternal-fetal cardiac coupling: a scoping review" is out of scope and it not technically sounded. writting formate is not valid and given figure 1 is not in good quality.

Thank you for sharing your feedback on our manuscript. We have uploaded a higher-quality version of Figure 1 using the required format (.tiff). However, we believe that our manuscript is within the scope of the journal and have not received any indication from the editor otherwise. We hope that by revising the manuscript in accordance with the comments received from the other reviewers, you will find the manuscript improved. 

Reviewer 2: Thomas et al. presented a fascinating scoping review on maternal-fetal cardiac coupling using available literature available on biomedical literature search engines. As a basic medicine researcher, I think it would be worth publishing such an article in PlosOne because it is well prepared and the findings are exciting. The introduction and literature search method to search literature is thorough. However, several inconsistencies throughout the article have been noticed. Please find below a couple of comments and concerns that the authors should address.

Thank you for your feedback on our work. We appreciate your positive comments. Furthermore, thank you for pointing out the inconsistencies in the manuscript; you will find these addressed pointwise below. Corresponding changes in the manuscript are highlighted in yellow. We believe that addressing these comments has improved the overall quality and coherence of the manuscript.

1. In the abstract section line 50, it would be clearer if the author added some words saying they included all the articles available regardless of the publishing years.

Thank you for your comment on the abstract section. We revised the abstract to explicitly state that the review encompassed articles from all available years, ensuring transparency about the inclusion criteria. The change can be found on page 3, lines 49 - 51:

 ‘Filters were only set for language (English, Dutch, and German literature were included) and, not for year of publication.’

2. On page 7 (Line 120), using a regular citation format to register the review protocol would be better than the current DOI link.

Thank you for your suggestion. Please see the update made in line 120, page 7: 

‘… on Open Science Framework [23].’

3. On page 7 (Line 129), what criteria have been searched of a researcher to consider them to call them active researchers in the field of MFCC? It should be mentioned to avoid the biases of literature searches.

Thank you for your comment. Your concern about potential biases in the literature search is valid. We’ve further clarified our search procedure on page 7, lines 130 – 131:

‘Additionally, a search was performed of all the works published by the researchers of the included studies to identify any further work concerning MFCC. ’ 

4. On page 8 (Line 153), how were the duplicates checked? It should be clearly mentioned.

Thank you for your comment regarding the process of checking for duplicates on page 8, line 153 of the manuscript. We have included the relevant information to clarify this process on page 7, lines 127 – 128:

‘This platform was also utilized to automatically identify and eliminate duplicate studies identified across multiple databases.’

5. On page 14 (Line 265), avoiding words like “Please note that” would be better. The author needs to revise it.

Thank you for your suggestion to reduce the verbosity of the text. Changes to the manuscript can be found in lines 228 (page 12), 267 (page 15), 321 (page 17), and 353 (page 19). 

6. On page 14 (Line 353), what is the basis of the proposed hypothesis? Is it based on some logic or some reference articles? It could be mentioned here.

The hypothesis was initially presented by Van Leeuwen et al, 2009. We have added this reference in line 357: 

‘… simultaneously modulating both the maternal and the fetal HR [27].’

7. The article title should be in lowercase on page 27 (Line 551) in reference 37.

Thank you for bringing this to our attention, please see the updated reference (now reference 38, line 550). 

8. Lastly, I recommend the authors to use a reference management software package like EndNote or Mendeley if they did not use it for this manuscript.

Thank you for your suggestion, we have used the reference management software Zotero.

Reviewer 3: This is an important scoping review addressing the phenomenon of maternal-fetal cardiac coupling (MFCC) and its potential clinical implications. The review did not explain how the difference between the normal fetal heart rate (120 – 160 bpm) and the normal mother's heart rate (60 – 100 bpm) could influence maternal-fetal cardiac coupling. Specifically, the authors mentioned that synchronization was more prevalent at higher respiration rates, while mothers who exercised regularly had lower incidences of MFCC than their less-active controls. High respiration and regular exercise are associated with a faster heart rate, which could hypothetically result in a higher incidence of MFCC. However, the outcomes from these two situations are entirely the opposite, which raises the question of the effect of the difference between the mother and fetal heart rates.

Thank you for your positive feedback and for pointing out this gap in our manuscript. There are two studies in question here. In the first one (Van Leeuwen et al., 2009), mothers were asked to breathe at different respiratory rates. Researchers then compared the incidence of MFCC between the different respiratory rates, finding that MFCC is more prevalent at higher respiratory rates (and correspondingly, higher heart rates). The second study compared MFCC between mothers who exercised regularly and those who didn’t (Van Leeuwen et al., 2014). The researchers found that those who exercised regularly had lower incidences of MFCC compared to their less-active controls. Regular exercise is known to contribute to cardiovascular fitness and as such, this group had lower resting respiratory rates and heart rates. Therefore, the results of these two studies complement each other. 

The revised manuscript includes some modifications to clarify this matter (highlighted in yellow). Please refer to page 13, line 247: 

‘On the other hand, mothers who exercised regularly during pregnancy had lower incidences of MFCC than their less active controls [28].’

As well as on page 18, line 351: 

‘Similarly, MFCC was less common in pregnancies where mothers had higher cardiovascular fitness and correspondingly lower resting respiratory rate and HR [28].’

Reviewer 4: The manuscript reads well and provides detailed scoping review on the topic of MFCC. Further, it directs future studies on mechanisms and nature of MFCC that may have implications on determining fetal cardiac health. I think it fulfils the need of scoping reviews.

Thank you for your positive feedback on the manuscript. We appreciate your recognition of the manuscript's potential implications for future studies. By directing research towards exploring the mechanisms and nature of MFCC, the review aims at contributing to the knowledge and clinical relevance of MFCC to track fetal well-being and fetal development during pregnancy.

Reviewer 5: Dear author I have gone through your scoping review, I must say that a very nicely literature is searched and written that the reader could understand the background of the study. A clear discussion written.

Thank you for your kind words about our scoping review. We are glad to hear that you found the literature search comprehensive and the writing clear and understandable.

# Peer review: Decision Letter 1

**DOI:** 10.1371/journal.pone.0287245.r003

**Authors:** Ahmed Mohamed Maged

2 Jun 2023

Evidence and clinical relevance of maternal-fetal cardiac coupling: a scoping review.

PONE-D-22-34923R1

Dear Dr. Nichting,

We’re pleased to inform you that your manuscript has been judged scientifically suitable for publication and will be formally accepted for publication once it meets all outstanding technical requirements.

Within one week, you’ll receive an e-mail detailing the required amendments. When these have been addressed, you’ll receive a formal acceptance letter and your manuscript will be scheduled for publication.

An invoice for payment will follow shortly after the formal acceptance. To ensure an efficient process, please log into Editorial Manager at http://www.editorialmanager.com/pone/, click the 'Update My Information' link at the top of the page, and double check that your user information is up-to-date. If you have any billing related questions, please contact our Author Billing department directly at authorbilling@plos.org.

If your institution or institutions have a press office, please notify them about your upcoming paper to help maximize its impact. If they’ll be preparing press materials, please inform our press team as soon as possible -- no later than 48 hours after receiving the formal acceptance. Your manuscript will remain under strict press embargo until 2 pm Eastern Time on the date of publication. For more information, please contact onepress@plos.org.

Kind regards,

Ahmed Mohamed Maged, MD

Academic Editor

PLOS ONE

Additional Editor Comments (optional):

Reviewers' comments:

Reviewer's Responses to Questions

**Comments to the Author**

1. If the authors have adequately addressed your comments raised in a previous round of review and you feel that this manuscript is now acceptable for publication, you may indicate that here to bypass the “Comments to the Author” section, enter your conflict of interest statement in the “Confidential to Editor” section, and submit your "Accept" recommendation.

Reviewer #2: All comments have been addressed

Reviewer #4: All comments have been addressed

Reviewer #5: All comments have been addressed

**********

2. Is the manuscript technically sound, and do the data support the conclusions?

The manuscript must describe a technically sound piece of scientific research with data that supports the conclusions. Experiments must have been conducted rigorously, with appropriate controls, replication, and sample sizes. The conclusions must be drawn appropriately based on the data presented. 

Reviewer #2: Yes

Reviewer #4: Yes

Reviewer #5: Yes

**********

3. Has the statistical analysis been performed appropriately and rigorously? 

Reviewer #2: N/A

Reviewer #4: N/A

Reviewer #5: Yes

**********

4. Have the authors made all data underlying the findings in their manuscript fully available?

The PLOS Data policy requires authors to make all data underlying the findings described in their manuscript fully available without restriction, with rare exception (please refer to the Data Availability Statement in the manuscript PDF file). The data should be provided as part of the manuscript or its supporting information, or deposited to a public repository. For example, in addition to summary statistics, the data points behind means, medians and variance measures should be available. If there are restrictions on publicly sharing data—e.g. participant privacy or use of data from a third party—those must be specified.

Reviewer #2: Yes

Reviewer #4: Yes

Reviewer #5: Yes

**********

5. Is the manuscript presented in an intelligible fashion and written in standard English?

PLOS ONE does not copyedit accepted manuscripts, so the language in submitted articles must be clear, correct, and unambiguous. Any typographical or grammatical errors should be corrected at revision, so please note any specific errors here.

Reviewer #2: Yes

Reviewer #4: Yes

Reviewer #5: Yes

**********

6. Review Comments to the Author

Please use the space provided to explain your answers to the questions above. You may also include additional comments for the author, including concerns about dual publication, research ethics, or publication ethics. (Please upload your review as an attachment if it exceeds 20,000 characters)

Reviewer #2: Thank you for resolving the comments addressed by me. I do not have additional comments. I believe that the presented scoping review on maternal-fetal cardiac coupling would interest in this field.

Reviewer #4: The manuscript shows potential implications for future studies by directing research towards exploring the mechanisms and nature of MFCC. It is appreciated that the review aims at contributing to the knowledge and clinical relevance of MFCC to track fetal development during pregnancy.

Reviewer #5: (No Response)

**********

7. PLOS authors have the option to publish the peer review history of their article (what does this mean?). If published, this will include your full peer review and any attached files.

If you choose “no”, your identity will remain anonymous but your review may still be made public.

**Do you want your identity to be public for this peer review?** For information about this choice, including consent withdrawal, please see our Privacy Policy.

Reviewer #2: **Yes: **A.S.M. WALIULLAH

Reviewer #4: **Yes: **Shradha Wali

Reviewer #5: **Yes: **Aisha Wali

**********

# Editor report: Acceptance letter

**DOI:** 10.1371/journal.pone.0287245.r004

**Authors:** Ahmed Mohamed Maged

15 Jun 2023

PONE-D-22-34923R1 

Evidence and clinical relevance of maternal-fetal cardiac coupling: a scoping review. 

Dear Dr. Nichting:

I'm pleased to inform you that your manuscript has been deemed suitable for publication in PLOS ONE. Congratulations! Your manuscript is now with our production department. 

If your institution or institutions have a press office, please let them know about your upcoming paper now to help maximize its impact. If they'll be preparing press materials, please inform our press team within the next 48 hours. Your manuscript will remain under strict press embargo until 2 pm Eastern Time on the date of publication. For more information please contact onepress@plos.org.

If we can help with anything else, please email us at plosone@plos.org. 

Thank you for submitting your work to PLOS ONE and supporting open access. 

Kind regards, 

PLOS ONE Editorial Office Staff

on behalf of

Professor Ahmed Mohamed Maged 

Academic Editor

PLOS ONE
